# Hypoxia and metabolic inhibitors alter the intracellular ATP:ADP ratio and membrane potential in human coronary artery smooth muscle cells

**DOI:** 10.7717/peerj.10344

**Published:** 2020-11-10

**Authors:** Mingming Yang, Caroline Dart, Tomoko Kamishima, John M. Quayle

**Affiliations:** 1 Department of Cardiology, Zhongda Hospital, Medical School of Southeast University, Nanjing, People’s Republic of China; 2 Department of Cellular and Molecular Physiology, Institute of Translational Medicine, Liverpool, UK; 3 Department of Biochemistry, Institute of Integrative Biology, Liverpool, UK

**Keywords:** Metabolic inhibitor, Hypoxia, ATP, Membrane potential, Potassium channels

## Abstract

ATP-sensitive potassium (K_ATP_) channels couple cellular metabolism to excitability, making them ideal candidate sensors for hypoxic vasodilation. However, it is still unknown whether cellular nucleotide levels are affected sufficiently to activate vascular K_ATP_ channels during hypoxia. To address this fundamental issue, we measured changes in the intracellular ATP:ADP ratio using the biosensors Perceval/PercevalHR, and membrane potential using the fluorescent probe DiBAC_4_(3) in human coronary artery smooth muscle cells (HCASMCs). ATP:ADP ratio was significantly reduced by exposure to hypoxia. Application of metabolic inhibitors for oxidative phosphorylation also reduced ATP:ADP ratio. Hyperpolarization caused by inhibiting oxidative phosphorylation was blocked by either 10 µM glibenclamide or 60 mM K^+^. Hyperpolarization caused by hypoxia was abolished by 60 mM K^+^ but not by individual K^+^ channel inhibitors. Taken together, these results suggest hypoxia causes hyperpolarization in part by modulating K^+^ channels in SMCs.

## Introduction

When the oxygen supply cannot meet the demand of metabolically active tissues, the systemic arteries dilate, causing an increase in local blood flow. This phenomenon known as hypoxic vasodilation was first reported as far back as 1880 by Roy & Brown (1880). Arteries supplying organs with changeable metabolic requirements need to produce fast and graded contractile responses. Coronary arteries are one such example since they supply blood to one of the most metabolically active organs. Thus, the coronary circulation is adapted to provide high basal rates of blood flow that can further increase more than ∼5 fold in response to enhanced cardiac work, for example during exercise (Duncker & Bache, 2008; Levick, 2010). This direct and tight link between metabolic demand and blood supply is a hallmark of the coronary circulation. A number of parallel mechanisms to achieve this may exist, and interpretation of studies where one pathway is blocked has been problematic due to this redundancy (Duncker & Bache, 2008). Presumably, these multiple mechanisms act as a safety net, ensuring supply of blood is guaranteed. This is a reflection of the absolute importance of coronary metabolic vasodilation, and also why it still remains poorly understood nearly 140 years after the original publication by Roy & Brown (1880). With this caveat, two of the major hypotheses that have been advanced to account for coronary metabolic vasodilation are: (i) the release of vasodilator metabolites from cardiac myocytes (e.g., adenosine, K^+^ and H^+^) acting on vascular smooth muscle cells (VSMCs), and (ii) the direct vasodilatory effects of hypoxia on VSMCs. Of course, these possibilities are not mutually exclusive, but if the latter scenario plays an important role, then hypoxia and metabolic insult should affect VSMCs in vitro.

One possible pathway for the second hypothesis described above is that hypoxia causes the opening of ATP-sensitive potassium (K_ATP_) channels, leading to VSMC hyperpolarization, a closure of voltage-dependent Ca^2+^ channels (VDCCs) and relaxation (Daut et al., 1990). K_ATP_ channels are ideally placed to couple cellular metabolic state to membrane excitability. K_ATP_ channels are perhaps best known for glucose sensation in pancreatic β-cells where an increase in blood glucose level causes channel closure through cellular ATP production (or, more precisely, via a change in the ATP:ADP ratio) (Misler et al., 1992). The resulting β-cell membrane depolarization triggers Ca^2+^ entry through VDCCs and so insulin secretion. The anti-diabetic sulphonylurea drugs such as glibenclamide cause insulin secretion by directly inhibiting K_ATP_ channels in β–cells (Sturgess et al., 1985). In cardiac myocytes, the K_ATP_ channel opens during severe and prolonged anoxia due to reduced ATP and increased ADP levels. This reduces cellular excitability, so preventing action potential generation and cell contraction (Flagg et al., 2010). Ultimately this protects the myocardium from damage by preventing complete depletion of intracellular ATP, which would trigger cell death. In the vasculature, K_ATP_channel have diverse functions (Flagg et al., 2010; Quayle, Nelson & Standen, 1997). K_ATP_ channels are activated by vasodilators coupled to cAMP-dependent protein kinase (PKA) and are inhibited by vasoconstrictors coupled to PKC (Kubo, Quayle & Standen, 1997). The resulting membrane potential changes are important in the actions of vasodilators and vasoconstrictors. Co-localization of K_ATP_ channels and protein kinases within specific plasma membrane microdomains, termed caveolae, appears important for modulation (Davies et al., 2010; Sampson et al., 2007; Sampson et al., 2004). Importantly, vascular K_ATP_ channels are activated by the anti-anginal compound nicorandil, and are inhibited by the sulphonylureas.

Many studies using intact coronary artery or whole heart preparation have provided evidence for a role of K_ATP_ channels in hypoxic vasodilation. The first report was made by Daut et al. (1990), in which vasodilation induced by hypoxia was inhibited by glibenclamide using a Langendorf preparation in intact guinea-pig heart. It was suggested that hypoxic vasodilation occurred via activation of coronary artery K_ATP_ channels, probably as a consequence of the change in cellular nucleotide levels (Von Beckerath et al., 1991). The results of a large number of functional studies in animal models are summarized in recent reviews (Duncker & Bache, 2008; Flagg et al., 2010; Shimoda & Polak, 2011). However, we know of only two attempts to address this issue in human coronary arteries. In arteries isolated from the right atrial appendage, hypoxia caused dilation that was not dependent on surrounding cardiac myocytes or the presence of the endothelium (Lynch et al., 2006; Miura et al., 2003). In one study pharmacological inhibition of K_ATP_ channels using a sulphonylrea (glibenclamide) blunted the hypoxic response (Miura et al., 2003), whilst in the other study glibenclamide was without effect (Lynch et al., 2006). K_ATP_ channels have been identified in cultured human coronary artery SMCs (HCASMCs) but there have been no studies looking at metabolic or other regulation of these channels in single cells (Yoshida et al., 2004). Regarding the physiological role of the K_ATP_ channel, it is of interest that genetic knockout in mice results in hypertension, coronary artery vasospasm and sudden cardiac death (Chutkow et al., 2002a; Miki et al., 2002). The effects of knockout have been linked to Prinzmetal angina, although it is not known if K_ATP_ channel mutations cause this disease in humans.

Despite the functional studies cited above, the apparently simple yet crucial question whether hypoxia changes cellular nucleotide levels to a degree sufficient to activate vascular K _ATP_ channels has proved to be difficult to address (Flagg et al., 2010). K_ATP_ channels appear to be mostly closed at levels of ATP normally expected in cells, and cytochrome oxidase, the key oxygen sensitive enzyme involved in mitochondrial ATP generation, is fully saturated with oxygen unless oxygen drops to very low levels (<1 mmHg, essentially equivalent to anoxia). In contrast, hypoxic vasodilation can occur following relatively mild drops in oxygen levels (∼30 mmHg) (Duncker & Bache, 2008). Indeed, a major recent review on the subject states that it remains unclear what the adenosine nucleotide levels are in the VSMCs and whether cellular metabolism changes sufficiently to dynamically regulate VSMC K_ATP_ channel activity under physiological conditions (Flagg et al., 2010). K_ATP_ currents in pig coronary artery are activated by hypoxia (PO_2_∼35 mmHg) (Dart & Standen, 1995). In contrast, K_ATP_ currents in rat femoral artery are not activated by hypoxia but are activated during anoxia (PO_2_ < 1 mmHg) (Quayle et al., 2006). This difference may reflect origin of artery (i.e., species, vascular bed, small vs large artery).

In this study, we exploited fluorescent probes (Perceval/PercevalHR) to directly measure changes in cellular nucleotide levels caused by hypoxia and metabolic inhibitors in real-time in HCASMCs. Changes in membrane potential were also measured, and effects of various K^+^ channel inhibitors were investigated. We report that hypoxia and metabolic inhibition cause membrane hyperpolarization at least in part through the activation of K^+^ channels.

## Material and Methods

### Cell culture

HCASMCs (Cat no. C-12511) were obtained from Promocell and maintained in smooth muscle growth medium 2 supplemented with smooth muscle growth medium 2 supplement mix (Promocell, Heidelberg, Germany) with a final supplement concentration of (per ml): 5% v/v fetal calf serum, 0.5 ng epidermal growth factor (recombinant human), 2 ng basic fibroblast growth factor (recombinant human) and 5 µg insulin (recombinant human). Penicillin/streptomycin (Thermo Fisher Scientific, Waltham, MA) was added to cell culture medium with a final concentration of 100 U/mL and 100 µg/mL, respectively. Cells were used between P7-P13. The expression of signature smooth muscle proteins, SMC α-actin, calponin and myosin heavy chain (MHC) was maintained for the duration of culture as detected by Western blot (WB) (Fig. S1) and immunocytochemistry (ICC) (Fig. S2).

### Subcultivation of HCASMCs

HCASMCs were grown in T75 cm^2^ flasks with 15 ml fully supplemented smooth muscle growth medium 2 in a 5% CO_2_ humidified incubator at a temperature of 37 °C. Cells were routinely passaged every 48–72 h when reaching about 70–80% confluency. Cell culture medium was aspirated, and the cells were washed with 15 ml pre-warmed Dulbecco’s Phosphate Buffered Saline (DPBS, no Ca^2+^/Mg^2+^, Cat no. 14190-144, Gibco) or HEPES Buffered Saline Solution (HepesBSS). Cells were then trypsinised with 1.5 ml versene (Gibco) containing 0.025% trypsin (Gibco) or 100 µl per cm^2^ Trypsin/EDTA Solution (0.04%/0.03%) for approximately 1 min. Enzyme was neutralized by addition of 8 ml fully supplemented cell culture medium or 100 µl per cm^2^ Trypsin Neutralizing Solution (TNS). HepesBSS, Trypsin/EDTA Solution and TNS were all contained in Promocell DetachKit (Cat no. C-41200). Cells were then re-suspended in fully supplemented fresh cell culture medium and cultured in T75 cm^2^ flasks at a volume of 15 ml.

### Western blotting

Proteins were separated according to their size by 10% sodium dodecyl sulphate polyacrylamide gel electrophoresis (SDS-PAGE). Separated proteins were transferred from polyacrylamide gel onto nitrocellulose membrane (Amersham Hybond ECL, Cat no. RPN303D) for western blot analyses. Ponceau S solution (Sigma, Cat no. P7170) for 5 min to check the transfer efficiency of the protein. Membranes were blocked in 1% w/v non-fat powdered milk (Marvel) before incubation with the following primary and secondary antibodies: anti α-actin (Sigma-Aldrich, Cat no. A2547) at 500 fold dilution, anti calponin (Sigma-Aldrich, Cat no. C2687) at 500 fold dilution, anti actin (Sigma-Aldrich, Cat no. A4700) at 500 fold dilution and anti-mouse horseradish peroxidase (HRP)-conjugated secondary antibody (Fitzgerald Industries International, Cat no. 43C-CB1569-FIT) at 10,000 fold dilution. Protein bands were detected with ECL western blotting detection reagents (Amersham, Cat no. RPN2109). Bands were sized by referencing to the rainbow protein mass marker (Amersham full-range rainbow MW marker, Cat no. RPN800E).

### Immunocytochemistry

HCASMCs were fixed with 2% w/v paraformaldehyde in PBS (in mM: 2.7 KCl, 1.5 KH2PO4, 137 NaCl, 8.0 Na2HPO4, pH 7.4) for 10 min and then incubated in permeabilisation solution (0.1% v/v Triton X-100 in PBS, pH adjusted to 7.4 using 1 M NaOH) for 10 min at room temperature. Next, the cells were incubated with 200 µl antibody diluting solution (in mM: SSC containing 250 NaCl and 15 Na_3_Citrate, 2% v/v goat serum, 0.05% v/v Triton X-100, 1% w/v bovine serum albumin, pH 7.2) for 30 min at room temperature to block non-specific binding. Cells were then incubated overnight at 4 °C with the following primary antibodies: anti α-actin (Sigma-Aldrich, Cat no. A2547) at 300 fold dilution, anti calponin (Sigma-Aldrich, Cat no. C2687) at 300 fold dilution and anti MHC (Novocastra, Cat no. NCL-MHCs) at 300 fold dilution. On the following day, primary antibodies were indirectly stained using fluorescent Alexa Fluor 488-conjugated goat anti-mouse IgG secondary antibody (Molecular probes, Cat no. A-11029) at the dilution of 1:500 for 2 h at room temperature. A LSM510 multiphoton laser-scanning confocal microscope was used to visualize the labelled cells. Alexa Fluor 488 was excited at 488 nm using an argon-ion laser, and emission light was collected at 500–550 nm.

### Cell transfection

Plasmids encoding Perceval, PercevalHR and pHRed were obtained from Addgene (Cambridge, MA). HCASMCs expressing PercevalHR or pHRed were transiently transfected using either FuGENE6 (Promega UK, Southampton, UK) or Promofectin (Promocell, Heidelberg, Germany) following manufacturer’s instruction. HCASMCs were seeded 24 h prior to transfection at a density of 1.25 × 10^5^ per well in 35 mm glass-bottom dishes (Greiner Bio-One, Gloucestershire, UK). The cells expressing Perceval and PercevalHR were used after 24–48 h of transfection, and cells expressing pHRed were also used after 24-48 h of transfection. The cells expressing PercevalHR based on a conventional 3rd generation lentivirus packaging system which has been described previously were used in PercevalHR in vitro calibration experiments (Yang et al., 2017).

### Confocal imaging

Experiments were carried out using either culture medium or physiological saline solution (PSS) containing [in mM]: 120 NaCl, 5 KCl, 1 MgCl_2_, 2 CaCl_2_, 0.42 Na_2_HPO_4_, 0.44 NaH_2_PO_4_, 24 NaHCO_3_, and 10 Glucose. Fluorescence signal of Perceval/PercevalHR and pHRed was measured with LSM510 (Carl Zeiss AG, Oberkochen, Germany), which allowed control of CO_2_, humidity and temperature while environmental O_2_ can also be changed to create hypoxic conditions. Manipulating O_2_ tension in the custom made micro-imaging chamber (atmospheric gas) was achieved by an O_2_ controller (# 0508.000, PeCon GmbH, Germany). This system has been specifically designed to image cultured cells under conditions of varying O_2_ tension, achieved by changing the O_2_ level in the atmosphere of the micro-imaging chamber. Images in the experiments of PercevalHR calibration were taken using LSM510 high speed multiphoton confocal laser scanning microscope (Carl Zeiss AG, Oberkochen, Germany). Software analysis of the data from LSM510 and LSM510 Multiphoton were carried out by region of interest analysis using AIM software, version 3.2 SP2 (Carl Zeiss AG).

### Measurement of intracellular ATP:ADP ratio, pH and membrane potential

Cells expressing Perceval or PercevalHR were excited with 488 nm light from an argon laser, and emission was collected at 500–550 nm. Cells expressing pHRed was excited at 458 nm, and emission signal over 575 nm was collected. To examine change in membrane potential, HCASMCs were plated into 35 mm glass-bottom dishes a day before experiments. Next day, cells were washed twice with 2 ml PSS, then incubated with 1 µM DiBAC_4_(3) dissolved in PSS for 15 min in a 37 °C/5%CO_2_ incubator. Cells were then used without washing the dye as the continued presence of the dye in the extracellular media was required for this experiment. Cells were excited with 488 nm and the emission signal was captured at 500–550 nm. The photon counts (F) from Perceval/PercevalHR, pHRed and DiBAC_4_(3) were normalized against the initial reading (F_0_) at the start of experiments and expressed as a ratio (relative fluorescence (F/F_0_)).

### Permeabilisation of HCASMCs with Staphylococcus aureus α-toxin

It is of high importance that the signals from biosensors are calibrated in vitro to validate that sensitivity and range are satisfactory for planned experiments. Furthermore, although Perceval and PercevalHR are supposed to detect ATP:ADP ratio, it has been also reported that their signals are independent of ADP (Li et al., 2013). Thus, attempts were made to calibrate the PercevalHR using solutions containing known concentration of ATP and ADP. To achieve this, HCASMCs have to be permeabilised in such a way that hydrophilic nucleotides can be introduced into the cells without loss of biosensor. Thus, permeabilisation with α-toxin was carried out for PercevalHR in vitro calibration. Preliminary experiments resulted in, however, loss of HCASMCs during solution exchange due to cell detachment from the glass. This was due to the excessive HCASMC contraction in the solution once cells were permeablised. The reason for this is unknown, but in order to minimize loss of cells, 35 mm glass-bottom dishes were coated with Poly D-Lysine and 10 µM wortmannin was added to solutions.

HCASMCs were washed with calibration solution A containing (mM) 110 KCl, 30 NaOH, 10 KCl, 10 HEPES, 10 EGTA, and 0.05 EDTA (pH adjusted to 7.2) to remove Ca^2+^. Cells were then further washed with calibration solution B containing (in mM) 140 KCl, 10 NaCl, 10 HEPES, 2 EGTA, and 0.05 EDTA. Next, Cells were permeabilised with calibration solution B containing 200 µg/ml α-toxin for about 1 h at room temperature. Finally, cells were washed with calibration solution B and in vitro calibration was carried out using calibration solution B containing required concentrations of nucleotides while keeping free Mg^2+^ constant at 0.5 mM (All calculations were done using MAXCHELATOR: http://maxchelator.stanford.edu/).

### Drugs

DiBAC_4_(3) was obtained from Thermo Fisher Scientific. Rotenone, antimycin, oligomycin B and carbonyl cyanide 3-chlorophenylhydrazone (CCCP) (all from Sigma-Aldrich, Dorset, UK) were dissolved in DMSO as 1, 1, 6 and 10 mM stock solutions, respectively. K^+^ channels inhibitors, glibenclamide (Sigma-Aldrich), penitrem A (Alomone labs, Jerusalem, Israel), tram34 (Sigma-Aldrich), apamin (Tocris Bioscience, Abingdon, UK) and XE991 (Tocris Bioscience) were also prepared in DMSO as 10, 1, 10, 1 and 10 mM stock solutions, respectively. Other chemicals were obtained from Sigma-Aldrich or VWR (Leicestershire, UK).

### Statistical analysis

When appropriate, values are expressed as a mean ± standard error of mean (SEM). N indicates number of cells unless otherwise stated. Statistical significance was evaluated with a paired Student’s *t*-test or with ANOVA with Tukey’s test for post hoc analysis using SPSS software package (version 20.0, IBM, New York, NY).

## Results

### In vitro calibration using α-toxin permeablized HCASMCs confirms PercevalHR detects ATP concentration over physiological range and is sensitive to intracellular ADP

Perceval is a fluorescent biosensor of adenylate nucleotides (Berg, Hung & Yellen, 2009) with T loop of ATP binding bacterial protein GlnK1 containing a yellow cpFP and circularly permuted monomeric Venus (cpmVenus). The ATP/ADP binding site is always occupied due to very high affinity, and the competition between ATP and ADP makes the sensor a reporter of intracellular ATP:ADP ratio (Berg, Hung & Yellen, 2009). PercevalHR is an improved fluorescent biosensor and is better tuned to sense the ATP:ADP ratios expected in healthy mammalian cells (Tantama et al., 2013; Tantama & Yellen, 2014).

**Figure 1:**
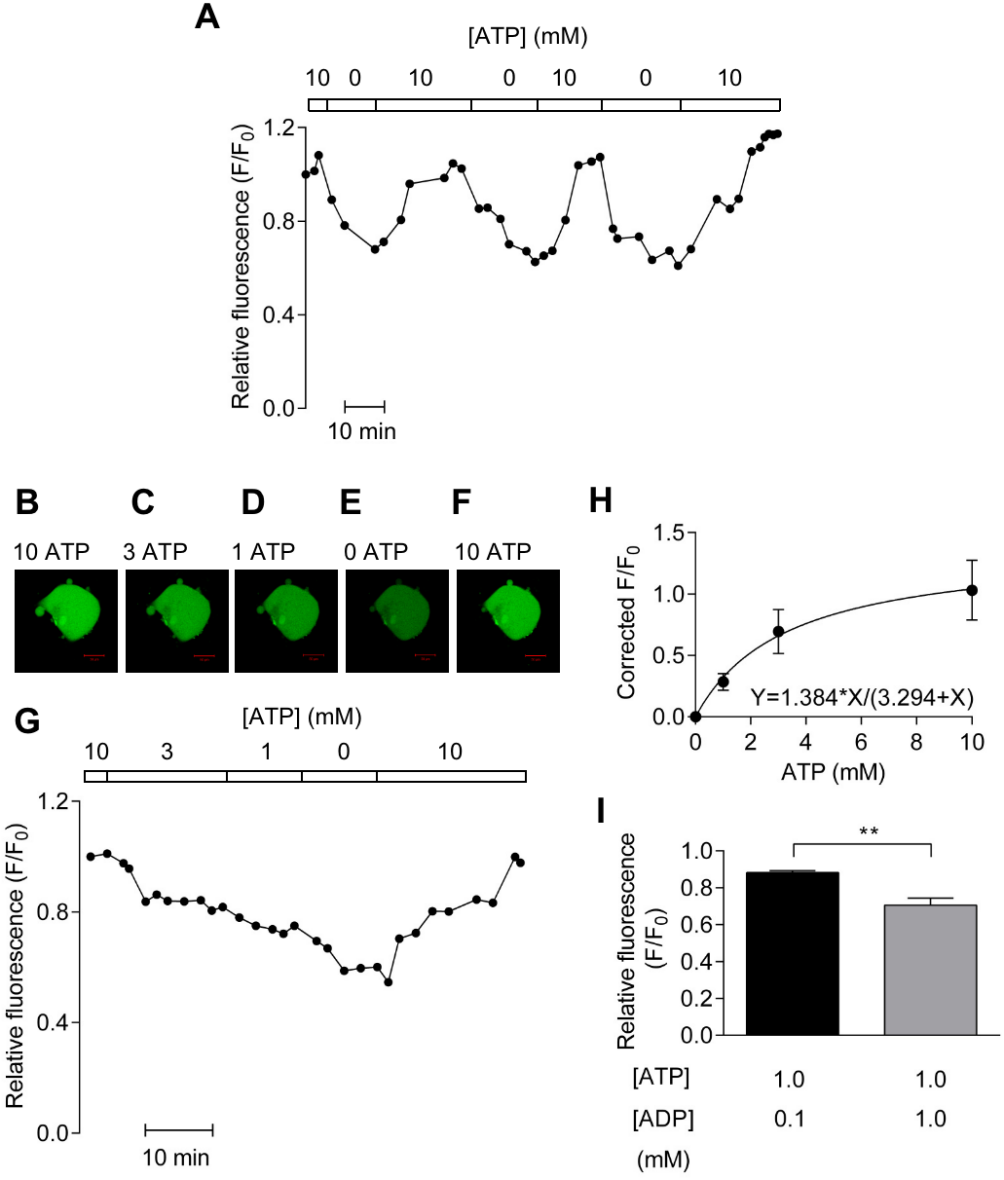
In vitro calibration of PercevalHR. (A) PercevalHR fluorescence from a single HCASMC permeabilized with *α*-toxin repeatedly exposed to 10 and 0 mM ATP. (B–G) PercevalHR fluorescence determined over physiological range of ATP concentrations (G) with confocal images of HCASMC (B–F). Scale bar is 50 µm. (H) Concentration-response relationship between ATP concentration and corrected fractional fluorescence (F/F_0_) with K_*D*_ of ∼3.3 mM ATP (*n* = 4 from four independent experiments). (I) Mean ± SEM of PercevalHR fluorescence determined with 0.1 mM ADP/1 mM ATP is significantly different from that with 1 mM ADP/1 mM ATP (*p* < 0.01, paired Student’s *t*-test, *n* = 5 from five independent experiments).

First, the sensitivity of PercevalHR to ATP over a physiological range and the effect of ADP on signal were assessed using α-toxin permeabilized HCASMCs. Figure 1A indicates that relative fluorescence (F/F_0_), determined in the presence of 10 mM ATP at the start of the experiment was reduced by a third with ATP-free solution. Upon the re-introduction of 10 mM ATP, relative fluorescence recovered to the previous level (Fig. 1A). The response of fluorescence probe to 10 mM and 0 mM ATP was stable and repeatable over 100 min, establishing that PercevalHR can report ATP level for a prolonged period.

Next, experiments were carried out to determine the K_D_ of PercevalHR in vitro using extracellular solutions containing 10, 3, 1 and 0 mM ATP. Figure 1B depicts a representative result showing confocal images of α-toxin-permeabilized HCASMCs expressing PercevalHR (Figs. 1B–1F) and the time-course of relative fluorescence change with varying concentrations of extracellular ATP (Fig. 1G). The original data show that the signal strength of PercevalHR was proportional to the concentration of extracellular ATP. When concentration–response curve was constructed, curve fitting showed a half maximal effect occurring at ∼3.3 mM of ATP (Fig. 1H).

As described earlier, one publication reported that Perceval is insensitive to ADP (Li et al., 2013). This is an important unresolved issue as some metabolically sensitive detectors such as K_ATP_ channels are regulated by not only absolute amount of ATP but also ADP (Quayle et al., 1994). We therefore studied the effect of intracellular ADP on PercevalHR fluorescence measured using 1 mM ATP in the presence of either 0.1 or 1 mM ADP. As shown in Fig. 1I, increasing ADP concentration from 0.1 mM to 1 mM reduced relative fluorescence significantly (*p* < 0.01) indicating that PercevalHR reports change in ATP:ADP ratio. Taken together, results obtained with permeabilised HCASMCs showed that PercevalHR can be used as a reporter of physiological ATP:ADP change.

### Removal of glucose from extracellular solution reduces the ATP:ADP ratio in HCASMCs

**Figure 2:**
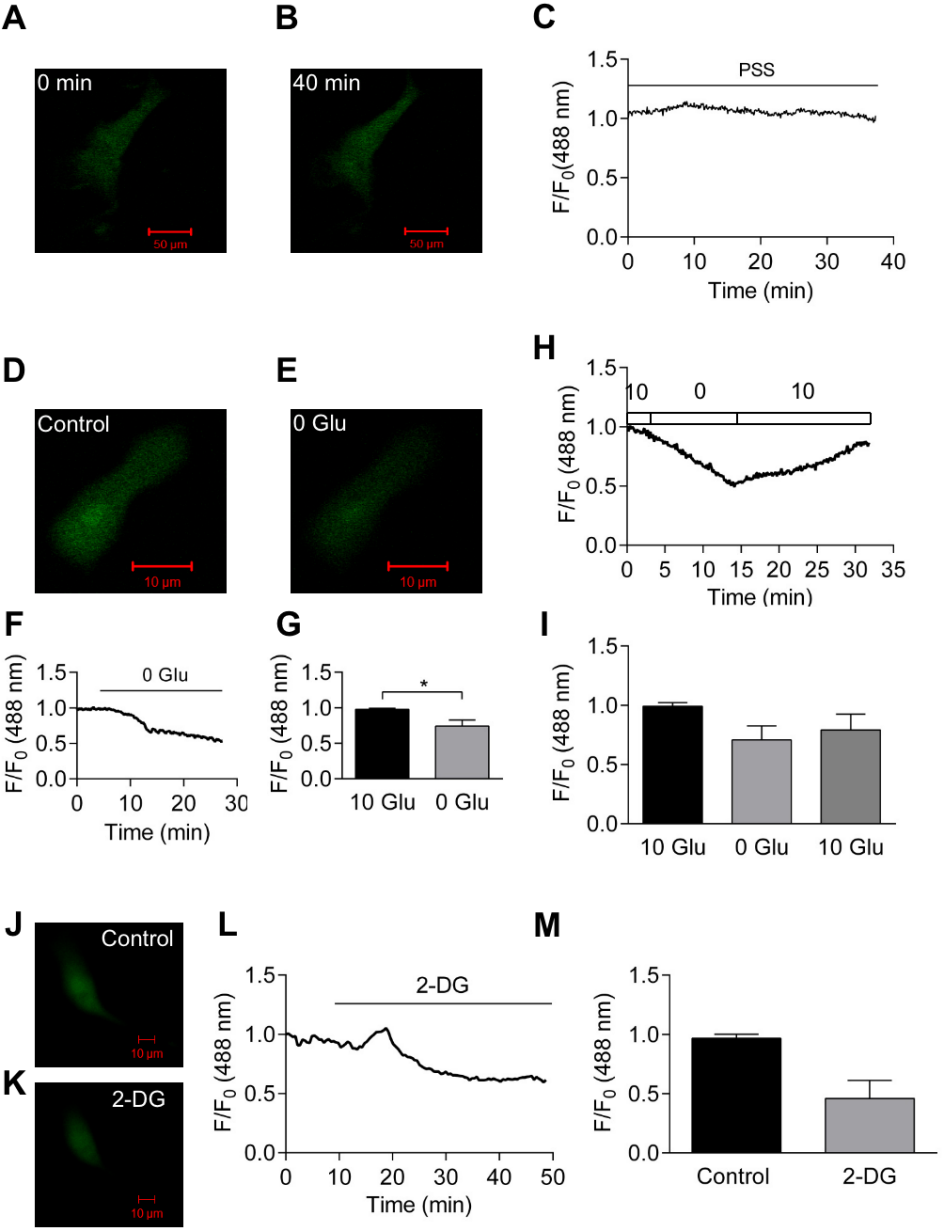
Effect of glucose removal on Perceval signal. (A–C) Perfusion with PSS for 40 min caused little change in fluorescence signal. (D–G) Images of HCASMC expressing Perceval before (D) and after glucose removal (E). The Perceval signal decreased after glucose removal (F). Fractional fluorescence just before (10 Glu) and after (0 Glu) glucose removal was significantly different (G, *p* < 0.05, paired Student’s *t*-test, *n* = 6 from 6 independent experiments). (H–I) Time course of fractional fluorescence caused by glucose removal and re-administration (H), and Mean ± SEM of fractional fluorescence for control, 0 mM glucose and 10 mM glucose (I, *n* = 3 from three independent experiments). (J–M) Images of HCASMC expressing Perceval at control condition (J) and after the application of 2-DG (K) with time course of fluorescence signal (L). Mean ± SEM of fractional fluorescence of Perceval was measured just before (control) and after administration of 2-DG (M, right panel, *P* = 0.112, paired Student’s *t*-test, *n* = 3 from three independent experiments).

Figures 2A–2C shows that Perceval fluorescence remained stable over a period of 40 min when the cell was perfused continuously with PSS. However, upon the removal of 10 mM extracellular glucose, the fluorescent signal dimmed (Figs. 2D–2E) and relative fluorescence decreased by almost 50% over 30 min (Fig. 2F). Removal of glucose significantly reduced relative fluorescence by 25.7 ± 8.3% (*p* < 0.05, *n* = 6, Fig. 2G). This reduction was not due to the bleaching of biosensor or loss of focus as the re-introduction of 10 mM extracellular glucose restored the signal (original trace, Fig. 2H; summary of 3 cells, Fig. 2I). When the glucose analogue, 2-deoxyglucose (2-DG, 5mM) that cannot be metabolized by the cell, was used, fluorescent signal became weaker (Figs. 2J–2L). Mean results from 3 cells showed that Perceval fluorescence signal was reduced by 54.0 ±15.3% after the application of 2-DG (*n* = 3, Fig. 2M). These results indicate that removal of extracellular glucose affects ATP:ADP ratio to a degree that is detectable by Perceval.

### Inhibition of oxidative phosphorylation causes a measurable decrease in cellular ATP:ADP ratio

**Figure 3:**
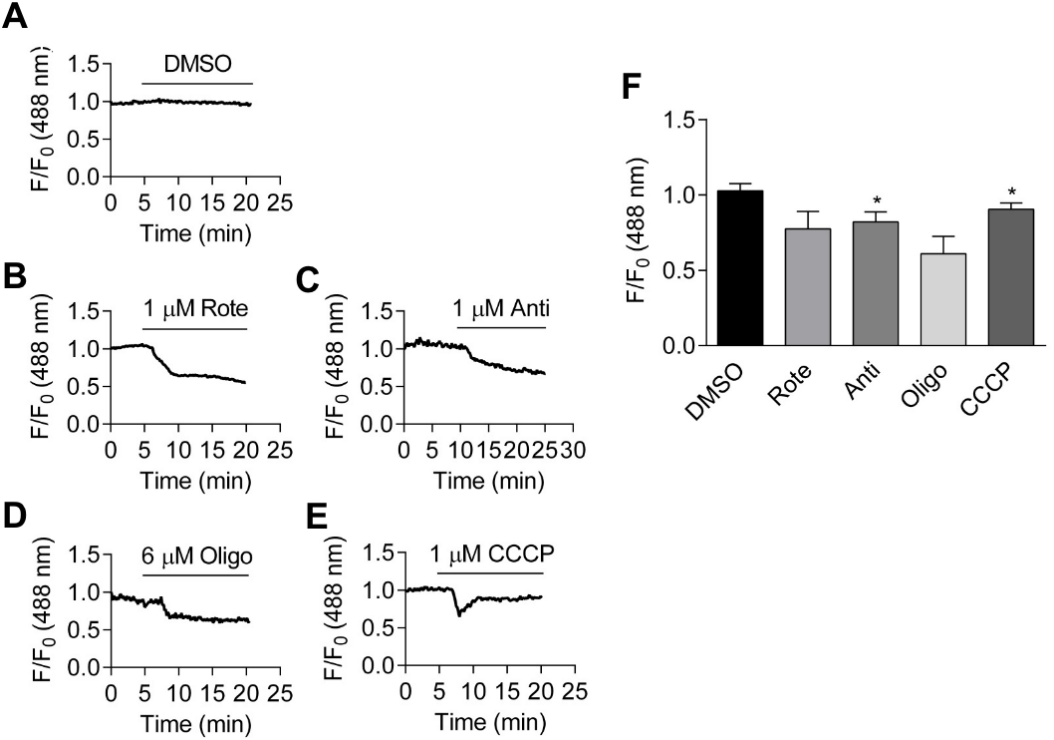
Effects of metabolic inhibitors on intracellular ATP:ADP ratio. (A–E): Time course of fractional fluorescence change from the cells treated with DMSO (A), 1 µM rotenone (B), 1 µM antimycin (C), 6 µM oligomycin (D) and 1 µM CCCP (E). (F): Mean ± SEM of fractional fluorescence after application of DMSO (*p* = 0.074, paired Student’s *t*-test, *n* = 4 from four independent experiments), rotenone (*p* = 0.111, paired Student’s *t*-test, *n* = 3 from three independent experiments), antimycin (*p* < 0.05, paired Student’s *t*-test, *n* = 4 from four independent experiments), oligomycin (*p* = 0.085, paired Student’s *t*-test, *n* = 3 from three independent experiments) and CCCP, measured at the peak (*p* < 0.05, paired Student’s *t*-test, *n* = 3 from three independent experiments).

ATP can be generated using extracellular glucose by oxidative phosphorylation and glycolysis pathways. Therefore, whether inhibition of oxidative phosphorylation affects PercevalHR signal was examined next in the cell culture medium. Oxidative phosphorylation was blocked by a number of approaches: inhibiting the electron transport chain with rotenone (1 µM), an inhibitor of mitochondrial complex I, or antimycin (1 µM), an inhibitor of mitochondrial complex III; inhibiting ATPase activity with oligomycin (6 µM), or through use of the uncoupling agent CCCP (1 µM). As shown in Figs. 3B–3E, application of metabolic inhibitors leads to a decrease in relative fluorescence. The effects of metabolic inhibitors, along with that of vehicle (DMSO), are summarized in Fig. 3F. When compared to the fluorescence ratio just before the administration of metabolic inhibitors, application of antimycin and CCCP induced significant reduction in ATP:ADP ratio signal (both *p* < 0.05).

Like other biosensors, Perceval may respond to changes in intracellular pH, which may accompany hypoxia (Berg, Hung & Yellen, 2009). Thus, the effect of hypoxia on the fluorescence of a pH sensor, pHRed, was also examined (Tantama, Hung & Yellen, 2011). pHRed responds robustly to intracellular alkalinization and acidification induced by the addition and subsequent removal of extracellular NH_4_Cl (20 mM) (Dart & Vaughan-Jones, 1992) (Fig. S3). There was little change in intracellular pH in response to metabolic interventions, as assessed by pHRed in cell culture medium (Fig. S4).

### Effect of hypoxia on cellular ATP:ADP ratio and pH

**Figure 4:**
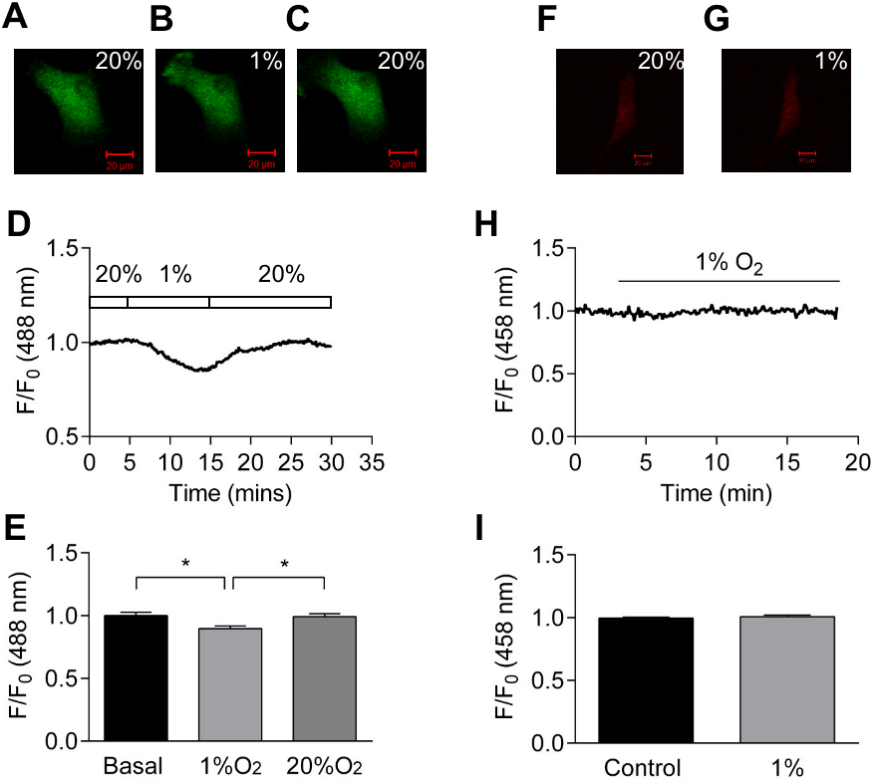
Effect of hypoxia on intracellular ATP:ADP ratio. (A–C) Confocal images of HCASMC expressing Perceval under control condition (A), after 10 minutes exposure to hypoxia (B), and recovery after re-oxygenation (C). (D) Time course of Perceval fractional fluorescence. (E) Exposure to hypoxia (1% O_2_) significantly changed Perceval fluorescence compared to control (basal) and recovery (20% O_2_) (*p* < 0.05, ANOVA with Tukey’s test for post hoc analysis, *n* = 5 from five independent experiments). (F–G) Confocal images of HCASMC expressing pHRed under control condition and during exposure to 1% O_2_. (H): Time course of fractional fluorescence. (I) Hypoxia caused little change in intracellular pH (bottom panel, *n* = 5 from five independent experiments).

Figure 4A-C show confocal images of HCASMC transiently transfected with Perceval under normoxia (20% oxygen), hypoxia (1% O_2_), and following the restoration of oxygen (20%). Fractional ATP:ADP ratio was reduced by about 10% by hypoxia and recovered upon oxygen reintroduction (Fig. 4D). Exposure to hypoxia significantly reduced Perceval signal by 10.3 ± 2.1%, and the change was reversible (*p* < 0.05, *n* = 5, Fig. 4E). There was no change in Perceval signal when mild hypoxia (10% and 5% O_2_) was tested (Fig. S5). pHRed however detected no change in intracellular pH after exposure to hypoxia (Figs. 4F–4I), suggesting that the fluorescence change induced in Perceval was due to changes in the ATP:ADP ratio and not an artefact caused by shift in intracellular pH. All the experiments were conducted in cell culture medium.

### Identification of K^+^ channels setting resting membrane potential of HCASMCs

DiBAC_4_(3) is a slow-response potential-sensitive fluorescent probe (Apell & Bersch, 1987; Mustafa et al., 2011), and thus a useful tool for HCASMCs where changes in membrane potential are generally slow and steady. It is permeable to the plasma membrane, accumulating in the cytoplasm following a Nernst equilibrium distribution (Apell & Bersch, 1987). The anionic dye in the cell binds to intracellular proteins or to membranes, resulting in an enhanced fluorescence and also a red spectral shift (Epps, Wolfe & Groppi, 1994; Klapperstuck et al., 2009). Depolarization of membrane potential causes entry of the anionic dye into the cell, increasing the fluorescence signal. Hyperpolarization, on the other hand, results in a decrease in the signal. Compared to cationic carbocyanines, DiBAC_4_(3) is largely excluded from mitochondria due to their overall negative charge, and so largely measures changes in the plasma membrane potential (Apell & Bersch, 1987; Baczkó, Giles & Light, 2004). To establish that DiBAC_4_(3) can detect moderate changes in membrane potential in HCASMCs, initial experiments were designed to characterize the changes in the relative fluorescence (F/F_0_) using the extracellular solutions containing various concentrations of [K^+^]_o_ (5, 30, and 80 mM). Resting membrane potential, assumed to mainly reflect K^+^ equilibrium potential, can be calculated with the Nernst equation. A representative characterization experiment is depicted in supplementary data, showing that the relationship of relative fluorescence and predicted membrane potential, note that the cultured HCASMCs have a more depolarized membrane potential at rest (Fig. S6).

**Figure 5:**
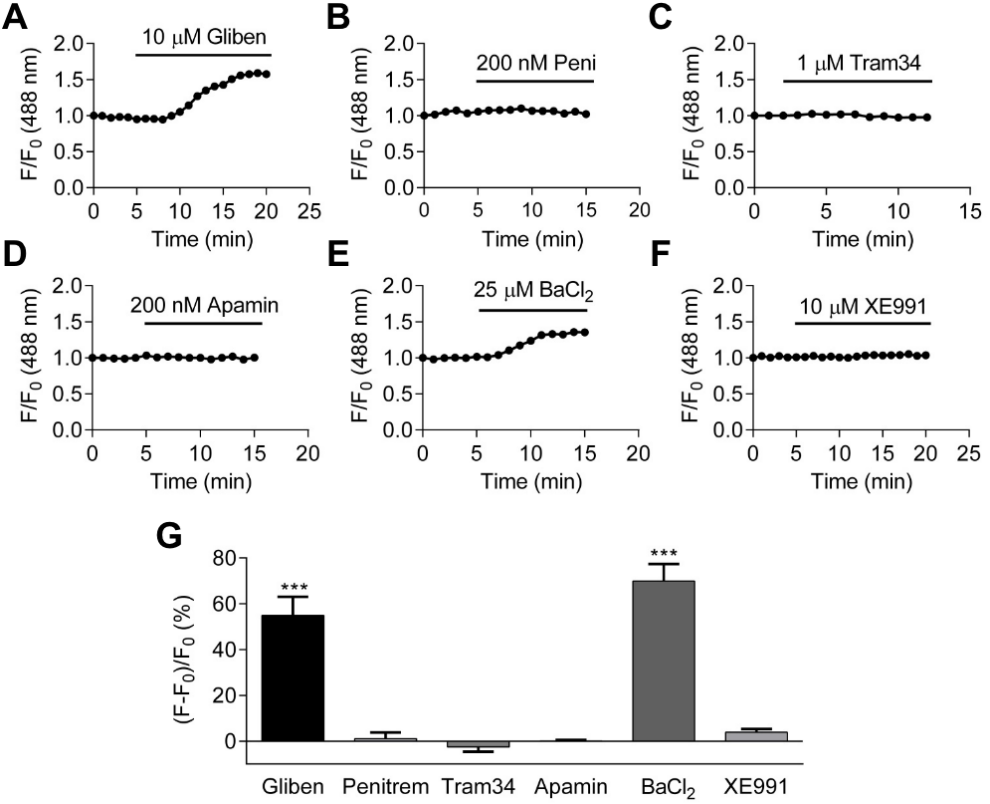
The identification of K^+^ channels in setting HCASMC resting membrane potential. (A–F) The change in the fractional fluorescence of DiBAC_4_(3) treated with 10 µM glibenclamide (A), 200 nM penitrem A (B), 1 µM tram34 (C), 200 nM apamin (D), 25 µM BaCl_2_ (E), and 10 µM XE991 (F). (G) A summary of percentchange in fractional fluorescence of DiBAC_4_(3) after application of K^+^ channel inhibitors: glibenclamide (*p* < 0.001, paired Student’s (*t*-test, *n* = 37 from three independent experiments), penitrem A *n* = 18 from two independent experiments), tram34 (*n* = 13 from 1 experiment), apamin (*n* = 32 from two independent experiments), BaCl_2_ (*p* < 0.001, paired Student’s *t*-test, *n* = 26 from two independent experiments), and XE991 (*n* = 14 from two independent experiments).

Like other excitable cells, HCASMCs express multiple types of K^+^ channels (Nelson & Quayle, 1995; Standen & Quayle, 1998). In order to investigate which K^+^ channel(s) are important in setting the resting membrane potential, a series of K^+^ channel inhibitors were applied. These included glibenclamide (10 µM), penitrem A (200 nM), tram34 (1 µM), apamin (200 nM), BaCl_2_(25 µM) and XE991 (10 µM) which are inhibitors of K_ATP_ channels, calcium-activated large, intermediate and small conductance potassium (BK_Ca_, IK_Ca_, SK_Ca_) channels, inward rectifying potassium (K_ir_) channels and voltage-gated potassium (K_v_7) channels respectively (Asano et al., 2012; Blatz & Magleby, 1987; Gluais et al., 2005; Ko et al., 2008; Mackie & Byron, 2008; Nelson & Quayle, 1995; Wulff & Castle, 2010). These inhibitors were used at concentrations thought to be selective for their respective class of channel (Alexander, Mathie & Peters, 2009). An increase in DiBAC_4_(3) signal (membrane depolarization) upon inhibition of a particular class of K^+^ channel would indicate that this channel is important in maintaining the resting membrane potential. Figs. 5A–5F show the representative time-course of relative fluorescence observed with different K^+^ channel inhibitors. The fractional fluorescence, determined as the difference between the relative fluorescence of DiBAC_4_(3) before and after drug application and expressed as percentage change is summarized in Fig. 5G. Application of glibenclamide and BaCl_2_ caused a significant change in fractional fluorescence (*p* < 0.001), suggesting that K_ATP_ and K_ir_ channels play major roles in setting resting membrane potential of HCASMCs.

### Effects of metabolic inhibitors and hypoxia on membrane potential

Of the K^+^ channels involved in setting the resting membrane potential of HCASMCs, K_ATP_ channels seem a likely candidate responsible for hypoxic vasodilation. Initial experiments using the K_ATP_ channel opener, pinacidil (10 µM), caused a significant decrease in DiBAC_4_(3) fluorescence (Fig. S7), suggesting that K_ATP_ channels have spare capacity to increase their open probability and/or active channel number from the resting condition. Therefore, the effect of metabolic insult and hypoxia on the DiBAC_4_(3) signal was examined.

**Figure 6:**
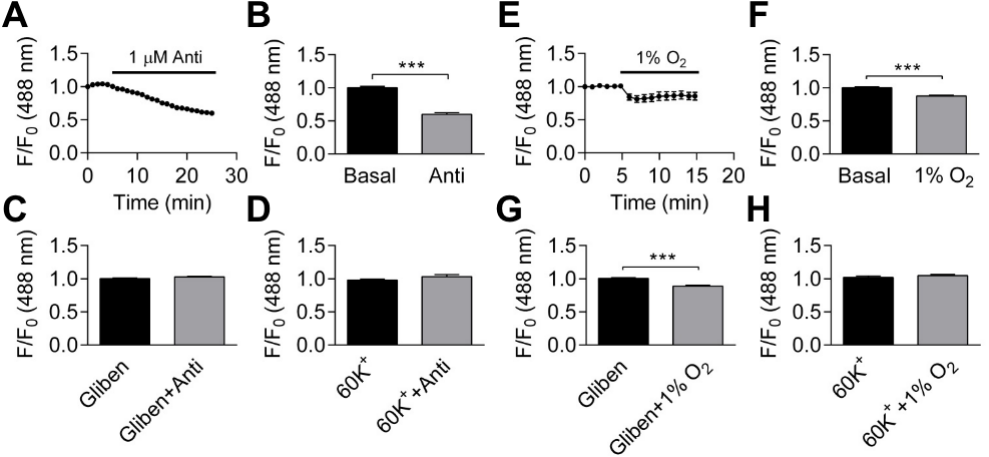
Effects of antimycin and hypoxia on membrane potential. (A–B) Application of 1 µM antimycin caused significant reduction in fractional fluorescence (*p* < 0.001, paired Student’s *t*-test, *n* = 25 from one experiment). (C–D) Pretreating the cells either with 10 µM glibenclamide (C, *n* = 22 from one experiment) or 60 K^+^ (D, *n* = 6 from one experiment) abolished the change induced by antimycin. (E–F) Hypoxia caused significant decrease in fractional fluorescence (*p* < 0.001, paired Student’s *t*-test, *n* = 72 from 11 independent experiments). (G–H): The effect of hypoxia was not blocked by 10 µM glibenclamide (G *p* < 0.001, paired Student’s *t*-test, *n* = 36 from four independent experiments), but hypoxia was ineffective with extracellular 60 K^+^ (H, *n* = 11 from two independent experiments, NS).

Inhibiting mitochondrial complex III with antimycin (1 µM) induced a significant decrease in DiBAC_4_(3) fluorescence (Figs. 6A–6B), which was blocked by glibenclamide (10 µM) or 60 K^+^ (Figs. 6C–6D), an indication that K_ATP_ channels may be involved. The effect induced by hypoxia on DiBAC_4_(3) fluorescence was highly significant (Figs. 6E–6F). The effect from hypoxia was blocked by 60 K^+^ (Fig. 6H), suggesting the involvement of K^+^ channels. However, exposure to hypoxia resulted in significant decrease in DiBAC_4_(3) fluorescence when in the presence of 10 µM pinacidil (*n* = 5), 10 µM glibenclamide (*n* = 36), 25 µM BaCl_2_ (*n* = 14), 100 nM IbTX (*n* = 17), 200 nM penitrem A (*n* = 14), and 10 µM glibenclamide plus 25 µM BaCl_2_(*n* = 25) (Fig. S8). These results indicate that K^+^ channels are likely to be involved in hypoxia-induced membrane hyperpolarization reported by DiBAC_4_(3), but classic K^+^ channel inhibitors, when applied singly or in combination in the case of glibenclamide plus BaCl_2_, failed to block the effect of hypoxia.

## Discussion

Many studies have reported that hypoxia activates various K^+^ channels in systemic arteries at different locations and of different species (Dart & Standen, 1995; Gebremedhin, Yamaura & Harder, 2008; Park et al., 2005; Park et al., 2007; Shimoda & Polak, 2011). Of these, K_ATP_ channels seem uniquely suited to act as the molecular targets, but to our knowledge, changes in nucleotide concentrations have not been measured in real time using VSMCs. The key issue addressed in the current paper was, therefore, whether the ATP:ADP ratio changes when HCASMCs are challenged by hypoxia and metabolic insults. This is the next step from our previous paper using the Seahorse technique showing that metabolic intervention resulted in a decrease, measured at the end point, in both cellular ATP and ATP:ADP ratio (Yang et al., 2017). The main finding of current paper is that 1% O_2_ hypoxia caused a significant decrease in ATP:ADP ratio in HCASMCs. Although this is somewhat surprising as we found that approximately half of ATP in these cells was produced by glycolysis that does not require oxygen (Yang et al., 2017), our present result shows that severe hypoxia can change the ATP:ADP ratio and that Perceval/PercevalHR can report such a change.

Compared to total ATP levels, the ATP:ADP ratio is arguably a more important parameter in cellular energy status. Perceval is originally constructed to monitor the changes in cellular ATP:ADP ratio (Berg, Hung & Yellen, 2009; Li et al., 2013). While Perceval, an original nucleotide biosensor, saturates at low ATP:ADP ratio (ATP:ADP <5) and is therefore tuned to extreme metabolic inhibition (Berg, Hung & Yellen, 2009), mammalian ATP:ADP ratios are estimated to range from 1 to 100 in healthy conditions (Traut, 1994; Veech et al., 1979). The improved sensor PercevalHR measures ATP:ADP ratio over the range expected in mammalian cells (Tantama et al., 2013; Tantama & Yellen, 2014). We report here that, using α-toxin permeabilized HCASMCs, K_D_ of PercevalHR for ATP is ∼3.3 mM (Fig. 1). It could be argued that, because the absolute amount of ATP, ADP and AMP can vary widely among and possibly within the cells, measurement of intracellular ATP:ADP and ATP:AMP ratios provide more reliable information of cellular metabolism (Berg, Hung & Yellen, 2009; Tantama & Yellen, 2014). When Perceval/PercevalHR were introduced to the cells to report intracellular ATP:ADP ratio, removal of glucose and inhibition of either glycolysis or oxidative phosphorylation with metabolic inhibitors all resulted in decreases in cellular ATP:ADP ratio (Figs. 2 and 3). Hypoxia induced a decrease in cellular ATP:ADP ratio (Fig. 4). The effect was not caused by a change in pH as parallel studies showed hypoxia exerted no change in pHRed signal (Fig. 4).

Although [ATP]_i_ is usually high enough to inhibit K_ATP_ channels, K_ATP_ channels may not sense global cytosolic ATP ([ATP]_c_). Rather, local concentrations of nucleotides in the microdomains close to K_ATP_ channels may be crucial, and it is conceivable that subcellular nucleotide concentrations dynamically change due to the activities of membrane ATPases and phosphotransfer enzymes. It has been reported that ATP binding site on K_ir_6.2 subunit is estimated to be ∼2 nm below the membrane and at the interface between adjacent K_ir_6.2 (Antcliff et al., 2005). Evidence has also suggested that local nucleotide level is modified when bulk ATP was transmitted over the diffusional barrier into the sub-membrane space due to the effect from metabolic enzymes (Flagg et al., 2010; Foster & Coetzee, 2016; Selivanov et al., 2004). AMP is suggested to regulate ATP:ADP ratio in the near membrane area by being involved in the phosphotransfer reactions mediated by *Adenylate kinase (*AK), resulting in a change sufficient to cause K_ATP_ channel opening (Carrasco et al., 2001). Taken together, ATP compartmentalization/intracellular concentration gradient may change sufficiently to regulate K_ATP_ channels whilst [ATP]_c_ is unchanged.

A biosensor to monitor pH was used in this study to ensure that the change in Perceval/PercevalHR signal was not an artifact due to acidification/alkalization of cells. A genetically encoded pH sensor, pHRed, made by mutagenesis of the red fluorescent protein mKeima, was used in this study (Tantama, Hung & Yellen, 2011). Metabolic inhibition of HCASMCs with 5 mM 2-DG when measured in bicarbonate buffered PSS or hypoxia when measured in cell culture medium induced no change in intracellular pH, and this is consistent with the results of other researchers (Imamura et al., 2009). Therefore, it seems safe to ascribe changes in Pervceval/PercevalHR signal to true changes in ATP:ADP. Indeed, intracellular pH of HCASMCs is rather well maintained as cells can handle pH changes via ion transporters on the cell membrane and by intracellular buffering (Boedtkjer, Praetorius & Aalkjaer, 2006; Madden, Keller & Kleinman, 2000). There is, however, reports showing that pH can change with metabolic inhibition (Imamura et al., 2009). In response to 2-DG, Kiang, McKinney & Gallin (1990) reported a ∼0.2 pH change in A-431 cells (Kiang, McKinney & Gallin, 1990), and Brown et al. (1991) found the same degree of pH change in isolated epithelial cells (Brown et al., 1991). It has been also reported that, in Neuro2A cells, metabolic inhibition by oligomycin and carbonyl cyanide 4-(trifluoromethoxy)phenylhydrazone (FCCP) showed no effect on cytosol pH whilst complete withdrawal of glucose acidified the cytosol (Tantama, Hung & Yellen, 2011). In the same study, it was shown that when pHRed was targeted to the mitochondrial matrix by COX8, it was reported an acidification of the mitochondrial matrix after the application of FCCP (Tantama, Hung & Yellen, 2011). Due to their crucial and multi-tasking function including ATP production cell signaling, cell differentiation, cell cycle and cell death, it would also be interesting to look at mitochondrial ATP specifically, especially when measured in combination with other substrates, such as Ca^2+^. However, it is beyond the scope of this study.

Many studies suggested that hypoxia causes vasodilation by decreasing [Ca^2+^]_i_. Some investigations indicated that hypoxia has a direct effect on Ca^2+^ channels while others have shown that hypoxia causes membrane potential hyperpolarization and a closure of VDCCs (Dart & Standen, 1995; Flagg et al., 2010; Francoobregon, Urena & Lopezbarneo, 1995; Welsh, Jackson & Segal, 1998). The observation in our lab that hypoxia inhibited Ca^2+^ oscillations in the presence of vasoconstrictors also indicates that Ca^2+^ handling itself is affected by hypoxia where the net effect would be reduction in [Ca^2+^]_i_ and muscle relaxation (Fig. S9, Table S1). Thus, the activation of K^+^ channels during hypoxic coronary vasodilation is aided by other mechanisms such as suppression of [Ca^2+^]_i_ transient during hypoxic vasodilation. Recent studies have also suggested that heme-protein myoglobin in VSMCs can produce nitric oxide (NO) through the reduction of endogenous nitrite under hypoxia and there leads to vasodilation, which regulates vasodilation via myoglobin-dependent NO generation and is independent of the well-known endothelial NO synthase (eNOS) (Hendgen-Cotta, Kelm & Rassaf, 2014; Totzeck et al., 2012).

At least two types of K^+^ channels are involved in setting resting membrane potential in HCASMCs, as application of 10 µM glibenclamide and 25 µM BaCl_2_ caused significant increase in DiBAC_4_(3) signal (Fig. 5). 25 µM BaCl_2_ also caused membrane potential depolarization in the presence of 10 µM glibenclamide (data not shown). Although Ba^2+^ inhibits multiple K^+^ channels including K_ATP_ channels at a higher concentration, at the concentration used in this study, BaCl_2_ should act as a specific blocker of K_ir_ channels (Nelson & Quayle, 1995; Quayle et al., 1993). The additive effect of glibenclamide and BaCl_2_ further supports the hypothesis that both K_ATP_ channels and K_ir_ channels regulate resting membrane potential in HCASMCs. Further characterization of K^+^ channels using selective activators and inhibitors showed that HCASMCs functionally express K_ATP_, BK_Ca_, and IK_Ca_/SK_Ca_ channels. In HCASMCs, changes in membrane potential caused by 10 µM NS1619 and 10 µM NS11021, both activators of BK_Ca_ channels, were not inhibited by 100 nM iberitoxin (IbTX) (data not shown), and this could be due to the presence of a specific β subunit in human coronary BK_Ca_ channels. A total of four types of modulatory β subunits have been identified, and in HCASMCs the majority of BK_Ca_ channels contain β1 subunit (KCNMB1) (Tanaka et al., 1997), which has higher sensitivity to Ca^2+^ and a lower sensitivity to IbTX (Dworetzky et al., 1996).

The most puzzling result regarding hypoxia effects on membrane potential was that none of the traditional K^+^ channel inhibitors eliminated the reduction in signal caused by hypoxia, although the effect of hypoxia was absent in high [K^+^]_o_. The lack of effect of traditional K^+^ channel inhibitors, when applied singly or as a combination of glibenclamide plus BaCl_2_, may be explained by the hypothesis that more than one class of K^+^ channel is involved, and so a combination of antagonists are required to block the hypoxia effect. Also, two-pore domain potassium (K2P) channels were not investigated in this study as there are no specific pharmacological inhibitors that can be used to dissect out the role of these channels. Moreover, the sensitivity of K^+^ channels to inhibitors may be reduced during hypoxia (Venkatesh, Lamp & Weiss, 1991). If the pharmacological tools are rendered less effective during hypoxia, then knocking out/down individual K^+^ channels using KO mice and siRNA might be useful to address some of these unanswered questions in future experiments.

Although cell culture is typically carried out at an atmospheric condition with an oxygen concentration of 20–21%, we are aware that cells in vivo reside under considerably lower oxygen tension, named physiologically relevant O_2_ concentration, also known as physioxia (Carreau et al., 2011). It has been reported that standard in vitro cultured cells experience a constant state of oxidative stress due to hyperoxic environment (Ferguson et al., 2018). Therefore, most of the contemporary experiments using in vitro cell culture are conducted with oxidatively stressed cells. Culture in hyperoxic conditions has conferred a protective resilience to many kinds of oxidative stressors, which is thought to be an artefact arising from the current culture leading to poor translation of investigations from in vitro to in vivo (Ferguson et al., 2018; Lin et al., 2020). On the contrary, the extent of antioxidant enzyme activities and redox homeostasis are examined to be closer to in vivo conditions if the cells are pre-conditioned under physioxia (Pastore et al., 2001; Shindo et al., 1994). Evidence has also pointed to a distinct pattern of protein expression between the two, and microRNAs are strong regulators of the transcriptome under hypoxic and physioxic conditions (Carreau et al., 2011). The different observations between hyperoxic and physioxic conditions should be well considered when looking at functional parameters in cells cultured in vitro, especially in experiments under various O_2_ concentrations. Furthermore, a range of physiologically-relevant O_2_ concentrations is suggested in order to reach a better understanding of the full scope of cellular responses as O_2_ level is different within a tissue. Although we have tried a range of O_2_ concentrations including 20%, 5% and 1% in this study, it is also crucial to conduct experiments in a variety of physiological O_2_ concentrations to make further understanding of the underlying K_ATP_ channel hypothesis. Moreover, suggestions have been made that chronic culture of mammalian cell culture under physioxia before exposure to experimental stimuli should be incorporated into standard protocols, which have been proved to be relatively easy and inexpensive to achieve (Ferguson et al., 2018).

## Conclusions

Hypoxic vasodilation is crucial to the regulation of human coronary blood flow, but underlying cellular and molecular mechanisms are unknown. The current paper provides evidence that direct opening of K^+^ channels of HCASMCs occurs in metabolic and hypoxic coronary vasodilation. Combined with the reports that altered Ca^2+^ and K^+^ channel function may be important in the coronary artery vascular changes seen in angina, diabetes and metabolic syndrome (Borbouse et al., 2010; Chutkow et al., 2002b; Dick & Tune, 2010; Miki et al., 2002; Miura et al., 2003), elucidation of detailed mechanisms underpinning hypoxic vasodilation will aid in development of therapeutic strategies to treat diseases associated with hypoxia/ischemia by inducing local vasodilation.

##  Supplemental Information

10.7717/peerj.10344/supp-1 Supplemental Information 1 WB of α-SMA and calponin in HCASMCs (A) α-SMA in HCASMC lysate from P7-P13, single band at 42 kDa, 20 minutes exposure. (B) Calponin in HCASMC lysate from P7-P13, with a primary band at 34 kDa, 20 minutes exposure. (C) Actin (re-blotting) in HCASMC lysate from P7-P13, single band at 42 kDa, 15 minutes exposure. All primary and secondary antibodies were diluted in TBST containing 5% non-fat powdered milk. Endothelial cell (EC) lysate was used as negative control.

10.7717/peerj.10344/supp-2 Supplemental Information 2 ICC of α-SMA, calponin and MHC expression. Confocal images of HCASMCs labelled with primary antibodies (anti-α-SMA, anti-calponin and anti-MHC) and AF488-conjugated secondary antibodies. Scale bar is 50 µm.

10.7717/peerj.10344/supp-3 Supplemental Information 3 pHRed reports changes in cellular pH. HCASMC transfected with pHRed before (left) and after (middle) application of NH_4_Cl with wash out (right) with fractional fluorescence time course and summary of 3 cells.

10.7717/peerj.10344/supp-4 Supplemental Information 4 Effect of metabolic interventions on intracellular pH. Top panel shows the time course of fractional fluorescence change from the cells treated with DMSO, 1 µM rotenone, and 6 µM oligomycin. Bottom panel shows Mean ± SEM of fractional fluorescence after application of DMSO (*p* < 0.05, *n* = 23), rotenone (*p* < 0.05, *n* = 5), and oligomycin (*p* < 0.05, *n* = 5).

10.7717/peerj.10344/supp-5 Supplemental Information 5 Effect of 10% and 5% O_2_ on Perceval signal. A: Time course of fractional fluorescence of Perceval showed little change during exposure to 5% O_2_ followed by a significant decrease under 1% O_2_. B: Mean ± SEM of fractional fluorescence of ATP:ADP ratio signal under normoxia, 5% O_2_ and 1% O_2_ (*n* = 4). C: Time course of fractional fluorescence of Perceval showed little change during exposure to 10% O_2_. D: Mean ± SEM of fractional fluorescence of ATP:ADP ratio signal under normoxia and 10% O_2_ (*n* = 4).

10.7717/peerj.10344/supp-6 Supplemental Information 6 Characterization of DiBAC_4_[b](3) fluorescence A: Images of DiBAC_4_(3) loaded cells with 5 mM, 30 mM and 80 mM [K^+^]_*o*_. B: DiBAC_4_(3) fluorescence changes upon increasing concentrations of [K^+^]_*o*_. C: Relationship between changes in DiBAC_4_(3) fluorescence and E_*K*_, where the resting membrane potential of HCASMCs are assumed to be −60 mV. The solid red line is Nernst equilibrium potential E_*k*_ calculated at 5, 30 and 80 mM K ^+^ (*Y* = 0.02131∗*X* + 2.807, *R*^2^ = 0.971). (*n* = 3).

10.7717/peerj.10344/supp-7 Supplemental Information 7 Effects of K_*ATP*_ channel modulators on membrane potential Left panel shows application of 10 µM pinacidil caused hyperpolarization while subsequent addition of 10 µM glibenclamide increased the signal above the basal level. Right panel shows Mean ± SEM of fractional fluorescence before and after the application of pinacidil and glibenclamide (*n* = 10).

10.7717/peerj.10344/supp-8 Supplemental Information 8 Role of K^+^ channels in hypoxia induced hyperpolarization The effect of hypoxia on membrane potential in the presence of 10 µM pinacidil (A, *n* = 5), 10 µM glibenclamide (B, *n* = 36), 25 µM BaCl_2_ (C, *n* = 14), 10 µM glibenclamide plus 25 µM BaCl_2_ (D, *n* = 25), 100 nM IbTX (E, *n* = 17), 200 nM penitrem A (F, *n* = 14).

10.7717/peerj.10344/supp-9 Supplemental Information 9 Effect of hypoxia on Ca^2+^ [b]oscillations (A) *Ca*^2+^ oscillations induced by 20 ng/ml PDGF-BB reported by Fluo-4. Frequency and the peak of oscillation were stable. (B) Hypoxia caused attenuation of *Ca*^2+^ oscillations induced by 10 µM PGF2a.

10.7717/peerj.10344/supp-10 Supplemental Information 10 Effect of hypoxia on calcium oscillations Effects of hypoxia (1% O2) on amplitude (A), frequency (F), AxF and Area under curve (AUC) of *Ca*^2+^ oscillations induced by PDGF (*n* = 7), PGF2 *α* (*n* = 6), U46619 (*n* = 17) and 60 K^+^ (*n* = 3).

10.7717/peerj.10344/supp-11 Supplemental Information 11 Figure 1A Raw data

10.7717/peerj.10344/supp-12 Supplemental Information 12 Figure 1B Raw data

10.7717/peerj.10344/supp-13 Supplemental Information 13 Figure 1C Raw data

10.7717/peerj.10344/supp-14 Supplemental Information 14 Figure 1D Raw data

10.7717/peerj.10344/supp-15 Supplemental Information 15 Figure 2A Raw data

10.7717/peerj.10344/supp-16 Supplemental Information 16 Figure 2B left Raw data

10.7717/peerj.10344/supp-17 Supplemental Information 17 Figure 2B right Raw data

10.7717/peerj.10344/supp-18 Supplemental Information 18 Figure 2C bottom Raw data

10.7717/peerj.10344/supp-19 Supplemental Information 19 Figure 2C top Raw data

10.7717/peerj.10344/supp-20 Supplemental Information 20 Figure 2D middle Raw data

10.7717/peerj.10344/supp-21 Supplemental Information 21 Figure 2D right Raw data

10.7717/peerj.10344/supp-22 Supplemental Information 22 Figure 3A Anti Raw data

10.7717/peerj.10344/supp-23 Supplemental Information 23 Figure 3A CCCP Raw data

10.7717/peerj.10344/supp-24 Supplemental Information 24 Figure 3A DMSO Raw data

10.7717/peerj.10344/supp-25 Supplemental Information 25 Figure 3A Oligo Raw data

10.7717/peerj.10344/supp-26 Supplemental Information 26 Figure 3A Rote Raw data

10.7717/peerj.10344/supp-27 Supplemental Information 27 Figure 3B Raw data

10.7717/peerj.10344/supp-28 Supplemental Information 28 Figure 4A bottom Raw data

10.7717/peerj.10344/supp-29 Supplemental Information 29 Figure 4B bottom Raw data

10.7717/peerj.10344/supp-30 Supplemental Information 30 Figure 4A middle Raw data

10.7717/peerj.10344/supp-31 Supplemental Information 31 Figure 4B middle Raw data

10.7717/peerj.10344/supp-32 Supplemental Information 32 Figure 5A BaCl2 Raw data

10.7717/peerj.10344/supp-33 Supplemental Information 33 Figure 5A Peni Raw data

10.7717/peerj.10344/supp-34 Supplemental Information 34 Figure 5A Apamin Raw data

10.7717/peerj.10344/supp-35 Supplemental Information 35 Figure 5B Raw data

10.7717/peerj.10344/supp-36 Supplemental Information 36 Figure 5A Gliben Raw data

10.7717/peerj.10344/supp-37 Supplemental Information 37 Figure 5A Tram Raw data

10.7717/peerj.10344/supp-38 Supplemental Information 38 Figure 5A XE991 Raw data

10.7717/peerj.10344/supp-39 Supplemental Information 39 Figure 6A bottom left Raw data

10.7717/peerj.10344/supp-40 Supplemental Information 40 Figure 6A bottom right Raw data

10.7717/peerj.10344/supp-41 Supplemental Information 41 Figure 6A top left Raw data

10.7717/peerj.10344/supp-42 Supplemental Information 42 Figure 6A top right Raw data

10.7717/peerj.10344/supp-43 Supplemental Information 43 Figure 6B bottom left Raw data

10.7717/peerj.10344/supp-44 Supplemental Information 44 Figure 6B bottom right Raw data

10.7717/peerj.10344/supp-45 Supplemental Information 45 Figure 6B top right Raw data

10.7717/peerj.10344/supp-46 Supplemental Information 46 Figure 6B top left Raw data

10.7717/peerj.10344/supp-47 Supplemental Information 47 Actin, Raw data for Fig. S1

10.7717/peerj.10344/supp-48 Supplemental Information 48 Calponin, Raw data for Fig. S1

10.7717/peerj.10344/supp-49 Supplemental Information 49 Alpha-actin, Raw data for Fig. S1

10.7717/peerj.10344/supp-50 Supplemental Information 50 Fig. S3B Raw data

10.7717/peerj.10344/supp-51 Supplemental Information 51 Fig. S3C Raw data

10.7717/peerj.10344/supp-52 Supplemental Information 52 Figure S4A bottom Raw data

10.7717/peerj.10344/supp-53 Supplemental Information 53 Figure S4A top Raw data

10.7717/peerj.10344/supp-54 Supplemental Information 54 Figure S4B bottom Raw data

10.7717/peerj.10344/supp-55 Supplemental Information 55 Figure S4B top Raw data

10.7717/peerj.10344/supp-56 Supplemental Information 56 Figure S4C bottom Raw data

10.7717/peerj.10344/supp-57 Supplemental Information 57 Figure S4C top Raw data

10.7717/peerj.10344/supp-58 Supplemental Information 58 Figure S5A left Raw data

10.7717/peerj.10344/supp-59 Supplemental Information 59 Figure S5A right Raw data

10.7717/peerj.10344/supp-60 Supplemental Information 60 Figure S5B left Raw data

10.7717/peerj.10344/supp-61 Supplemental Information 61 Figure S6B right Raw data

10.7717/peerj.10344/supp-62 Supplemental Information 62 Figure S6B Raw data

10.7717/peerj.10344/supp-63 Supplemental Information 63 Figure S6C Raw data

10.7717/peerj.10344/supp-64 Supplemental Information 64 Figure S7 left Raw data

10.7717/peerj.10344/supp-65 Supplemental Information 65 Figure S7 right Raw data

10.7717/peerj.10344/supp-66 Supplemental Information 66 Figure S8 bottom left Raw data

10.7717/peerj.10344/supp-67 Supplemental Information 67 Figure S8 bottom middle Raw data

10.7717/peerj.10344/supp-68 Supplemental Information 68 Figure S8 bottom right Raw data

10.7717/peerj.10344/supp-69 Supplemental Information 69 Figure S8 top left Raw data

10.7717/peerj.10344/supp-70 Supplemental Information 70 Figure S8 top middle Raw data

10.7717/peerj.10344/supp-71 Supplemental Information 71 Figure S8 top right Raw data

10.7717/peerj.10344/supp-72 Supplemental Information 72 Figure S9A Raw data

10.7717/peerj.10344/supp-73 Supplemental Information 73 Figure S9B Raw data

10.7717/peerj.10344/supp-74 Supplemental Information 74 Figure S1 full length blots

## Additional Information and Declarations

### Data Availability

The following information was supplied regarding data availability: The raw measurements are available in the Supplementary Files.
