# Supplementary figures and images for: Hypoxia and metabolic inhibitors alter the intracellular ATP:ADP ratio and membrane potential in human coronary artery smooth muscle cells
Source: PeerJ. 2020 Nov 10;8:e10344. doi: 10.7717/peerj.10344 (PMC7664465; doi:10.7717/peerj.10344)

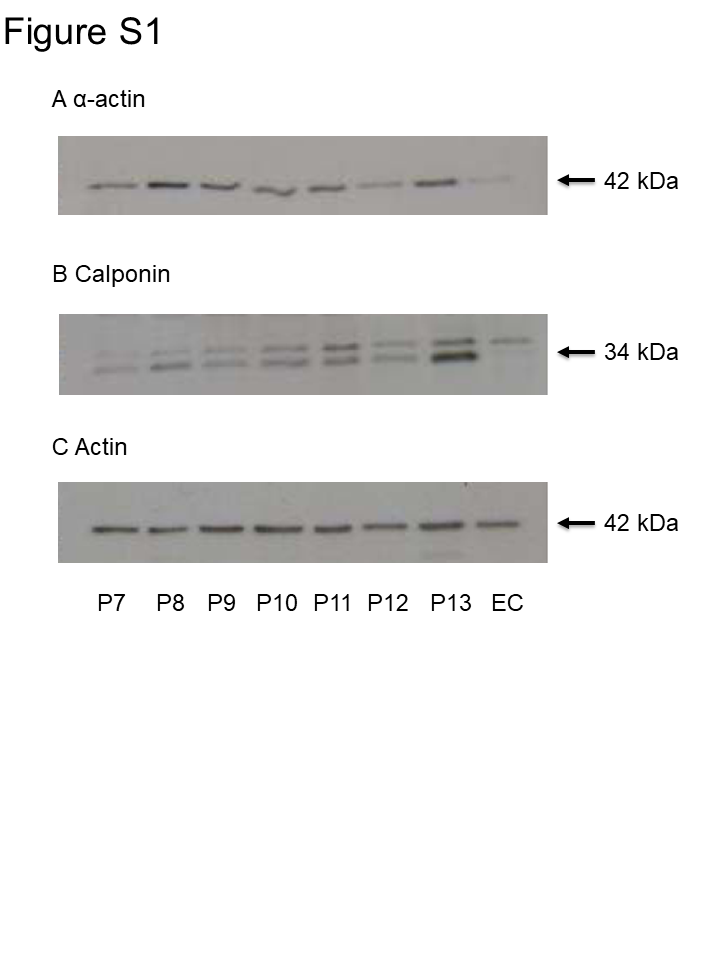

Supplement: Supplemental Information 1 — (A) α-SMA in HCASMC lysate from P7-P13, single band at 42 kDa, 20 minutes exposure. (B) Calponin in HCASMC lysate from P7-P13, with a primary band at 34 kDa, 20 minutes exposure. (C) Actin (re-blotting) in HCASMC lysate from P7-P13, single band at 42 kDa, 15 minutes exposure. All primary and secondary antibodies were diluted in TBST containing 5% non-fat powdered milk. Endothelial cell (EC) lysate was used as negative control. [file peerj-08-10344-s001.tif]

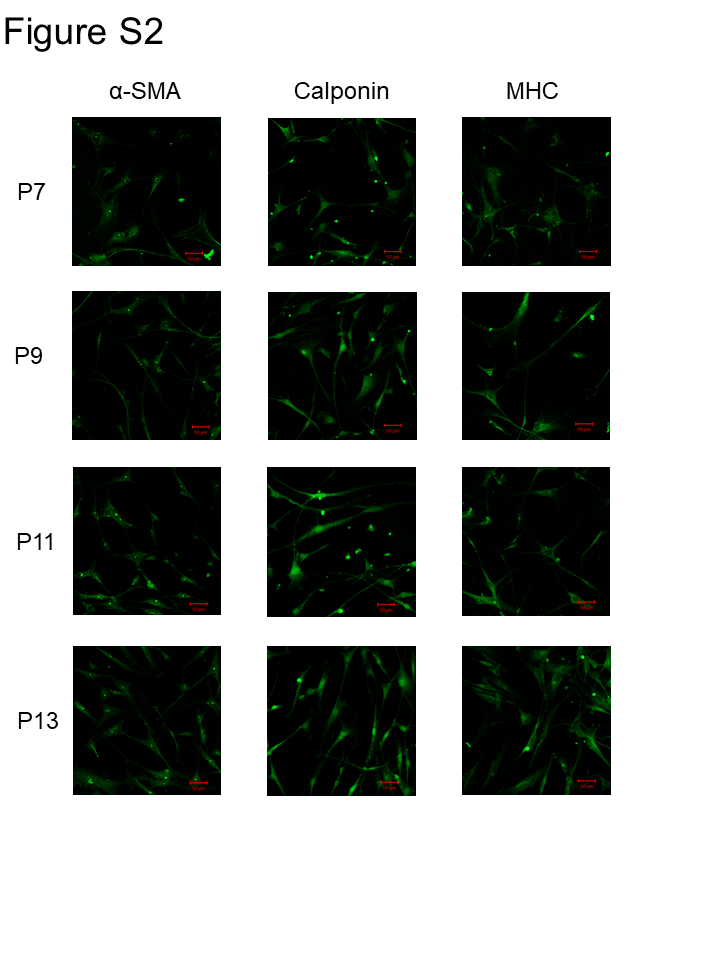

Supplement: Supplemental Information 2 — Confocal images of HCASMCs labelled with primary antibodies (anti-α-SMA, anti-calponin and anti-MHC) and AF488-conjugated secondary antibodies. Scale bar is 50 µm. [file peerj-08-10344-s002.tif]

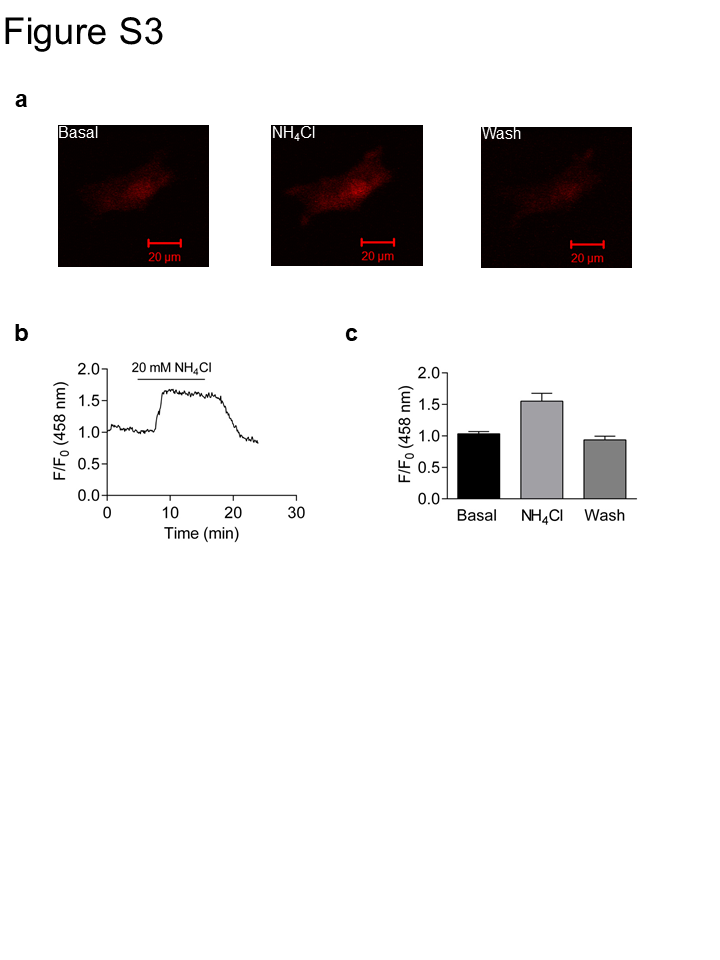

Supplement: Supplemental Information 3 — HCASMC transfected with pHRed before (left) and after (middle) application of NH4Cl with wash out (right) with fractional fluorescence time course and summary of 3 cells. [file peerj-08-10344-s003.tif]

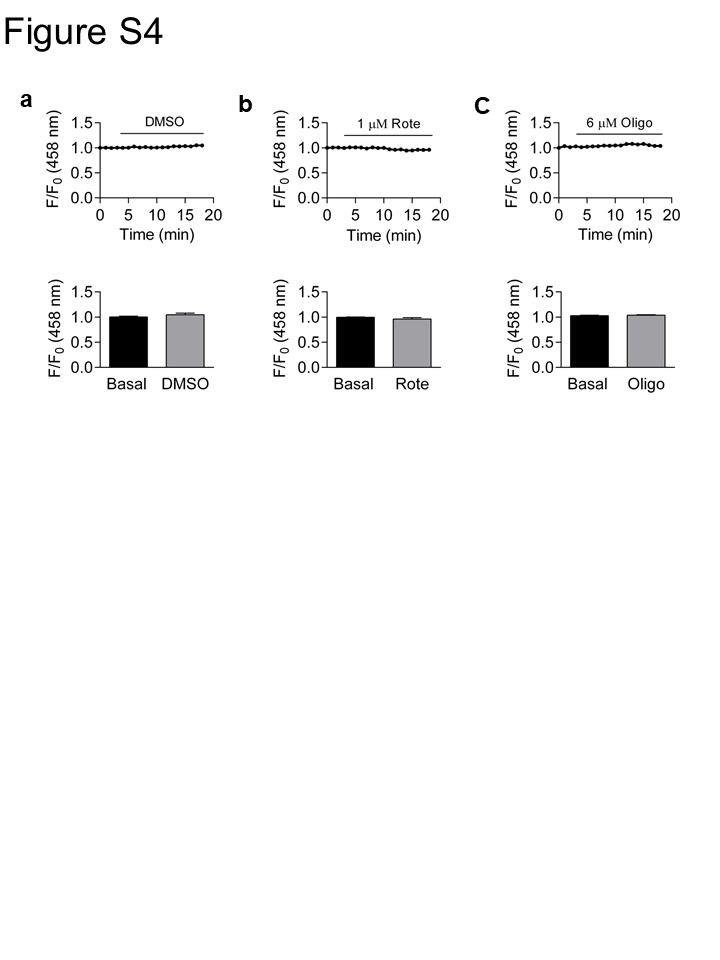

Supplement: Supplemental Information 4 — Top panel shows the time course of fractional fluorescence change from the cells treated with DMSO, 1 µM rotenone, and 6 µM oligomycin. Bottom panel shows Mean ± SEM of fractional fluorescence after application of DMSO (p < 0.05, n = 23), rotenone (p < 0.05, n = 5), and oligomycin (p < 0.05, n = 5). [file peerj-08-10344-s004.tif]

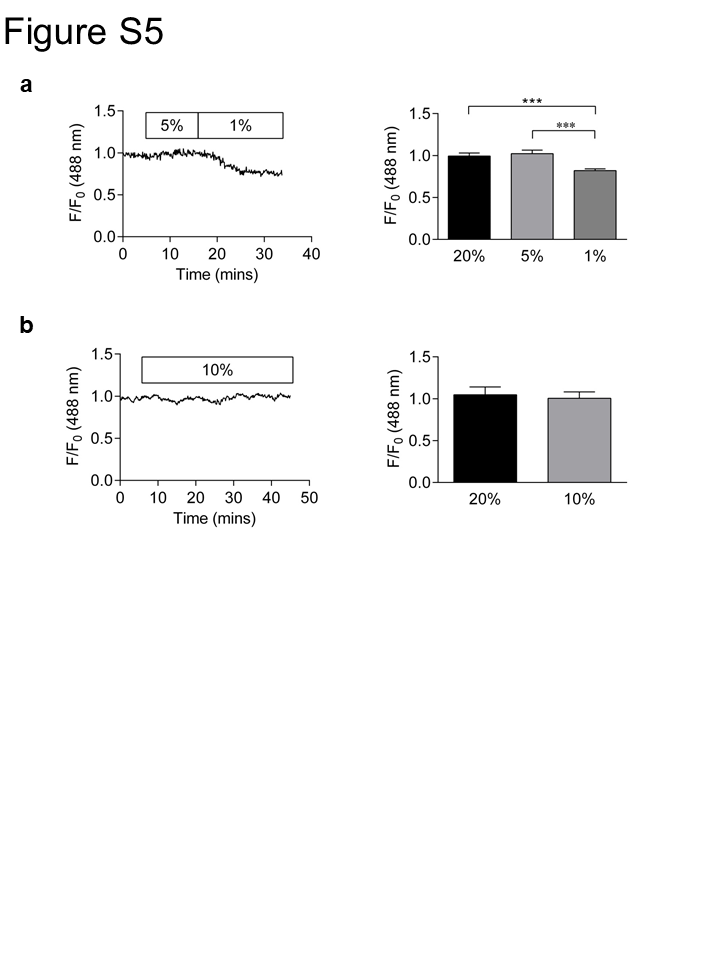

Supplement: Supplemental Information 5 — A: Time course of fractional fluorescence of Perceval showed little change during exposure to 5% O2 followed by a significant decrease under 1% O2. B: Mean ± SEM of fractional fluorescence of ATP:ADP ratio signal under normoxia, 5% O2 and 1% O2 (n = 4). C: Time course of fractional fluorescence of Perceval showed little change during exposure to 10% O2. D: Mean ± SEM of fractional fluorescence of ATP:ADP ratio signal under normoxia and 10% O2 (n = 4). [file peerj-08-10344-s005.tif]

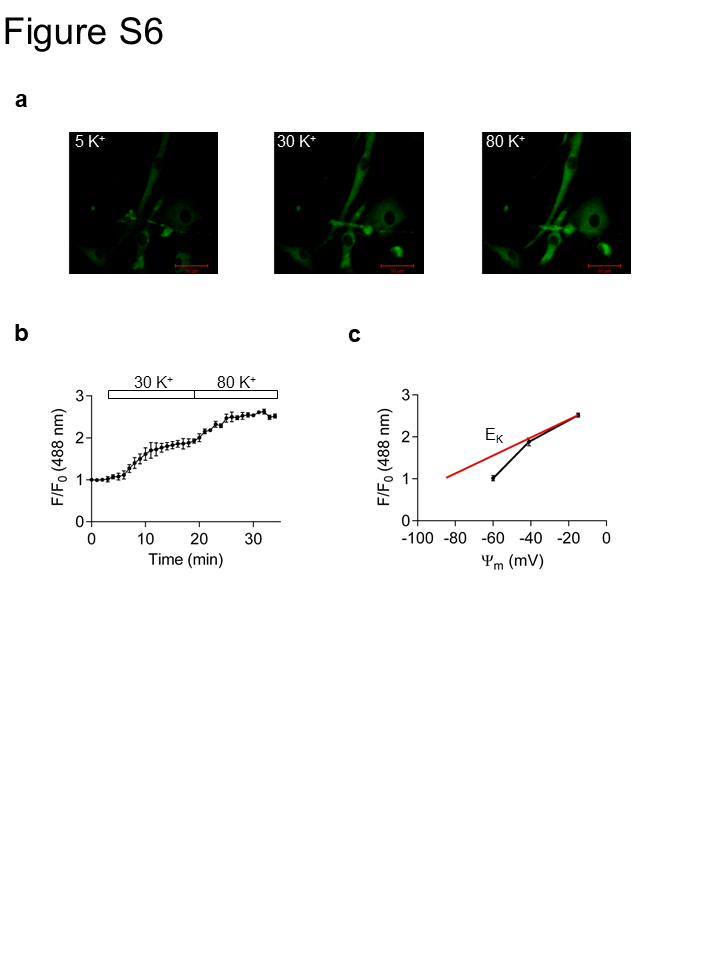

Supplement: Supplemental Information 6 — A: Images of DiBAC4(3) loaded cells with 5 mM, 30 mM and 80 mM [K+]o. B: DiBAC4(3) fluorescence changes upon increasing concentrations of [K+]o. C: Relationship between changes in DiBAC4(3) fluorescence and EK, where the resting membrane potential of HCASMCs are assumed to be −60 mV. The solid red line is Nernst equilibrium potential Ek calculated at 5, 30 and 80 mM K + (Y = 0.02131∗X + 2.807, R2 = 0.971). (n = 3). [file peerj-08-10344-s006.tif]

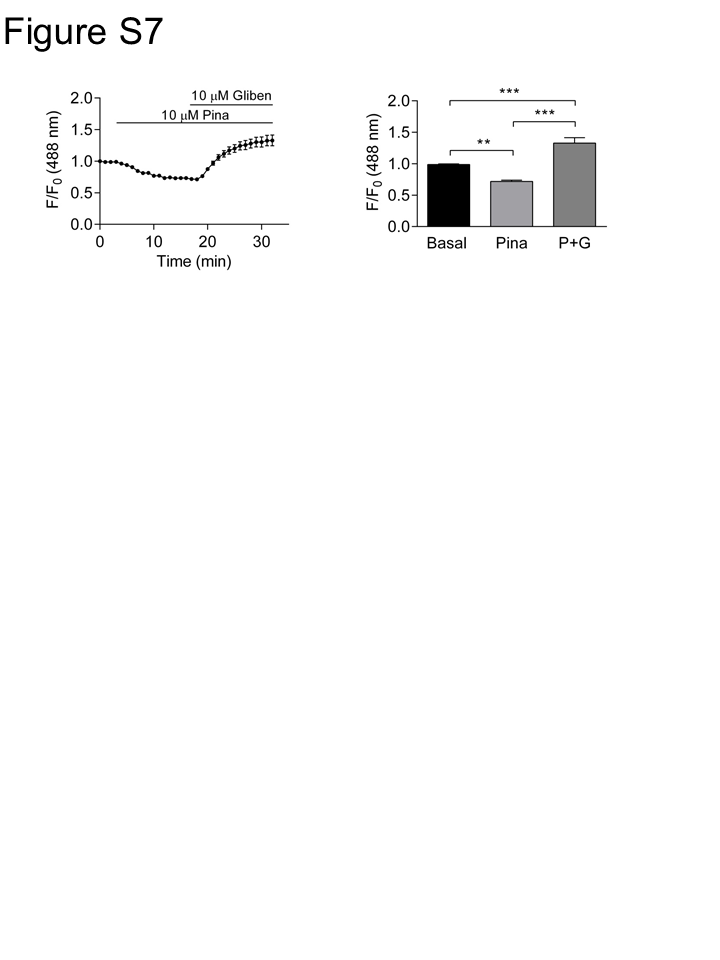

Supplement: Supplemental Information 7 — Left panel shows application of 10 µM pinacidil caused hyperpolarization while subsequent addition of 10 µM glibenclamide increased the signal above the basal level. Right panel shows Mean ± SEM of fractional fluorescence before and after the application of pinacidil and glibenclamide (n = 10). [file peerj-08-10344-s007.tif]

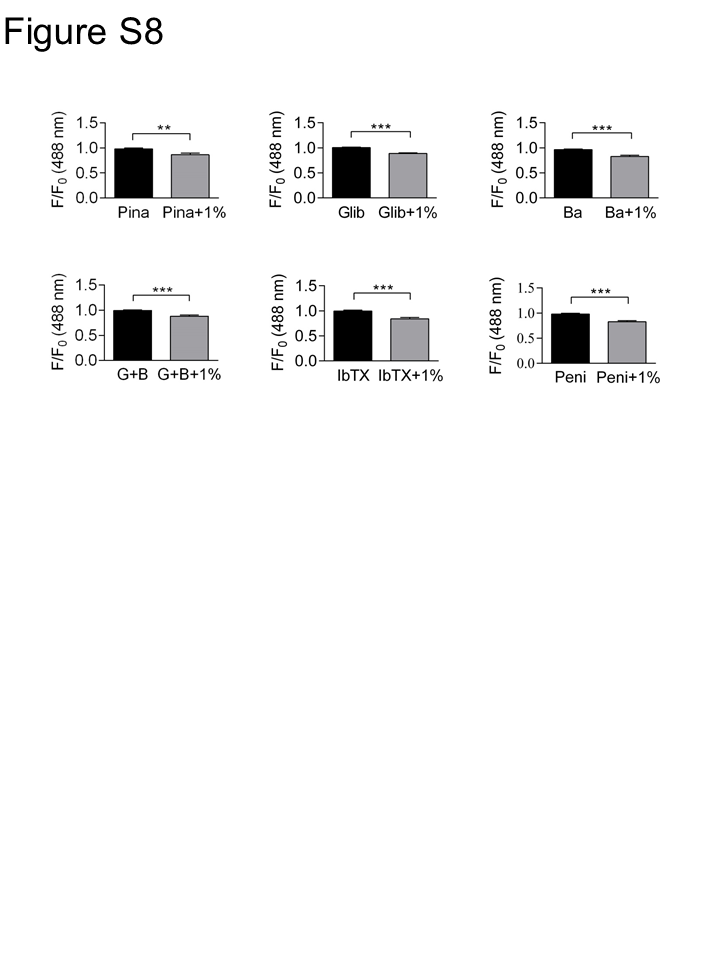

Supplement: Supplemental Information 8 — The effect of hypoxia on membrane potential in the presence of 10 µM pinacidil (A, n = 5), 10 µM glibenclamide (B, n = 36), 25 µM BaCl2 (C, n = 14), 10 µM glibenclamide plus 25 µM BaCl2 (D, n = 25), 100 nM IbTX (E, n = 17), 200 nM penitrem A (F, n = 14). [file peerj-08-10344-s008.tif]

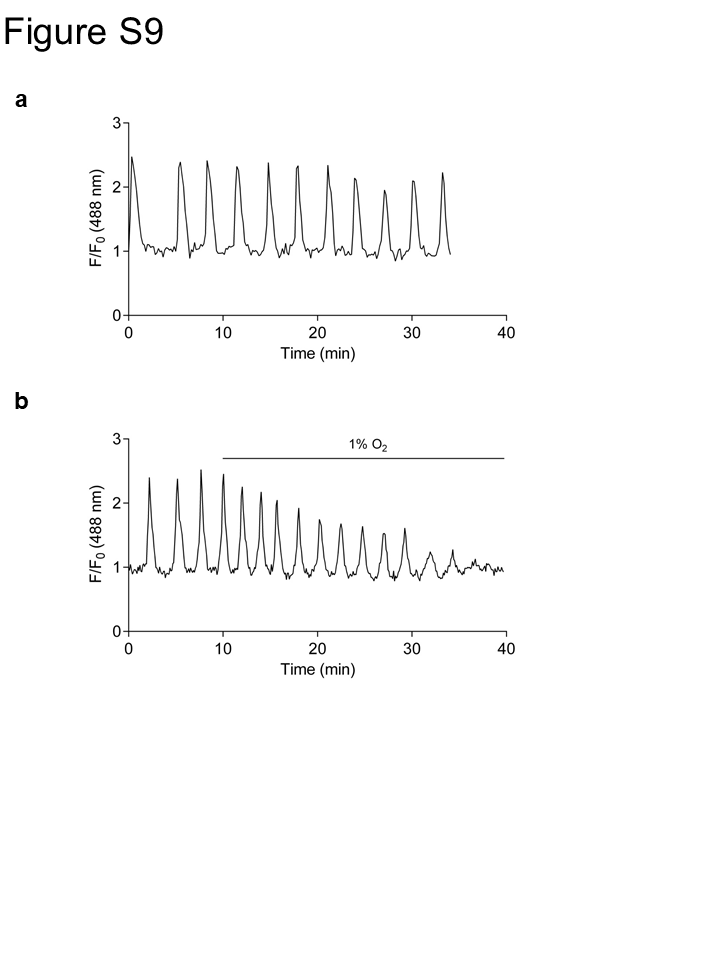

Supplement: Supplemental Information 9 — (A) Ca2+ oscillations induced by 20 ng/ml PDGF-BB reported by Fluo-4. Frequency and the peak of oscillation were stable. (B) Hypoxia caused attenuation of Ca2+ oscillations induced by 10 µM PGF2a. [file peerj-08-10344-s009.tif]

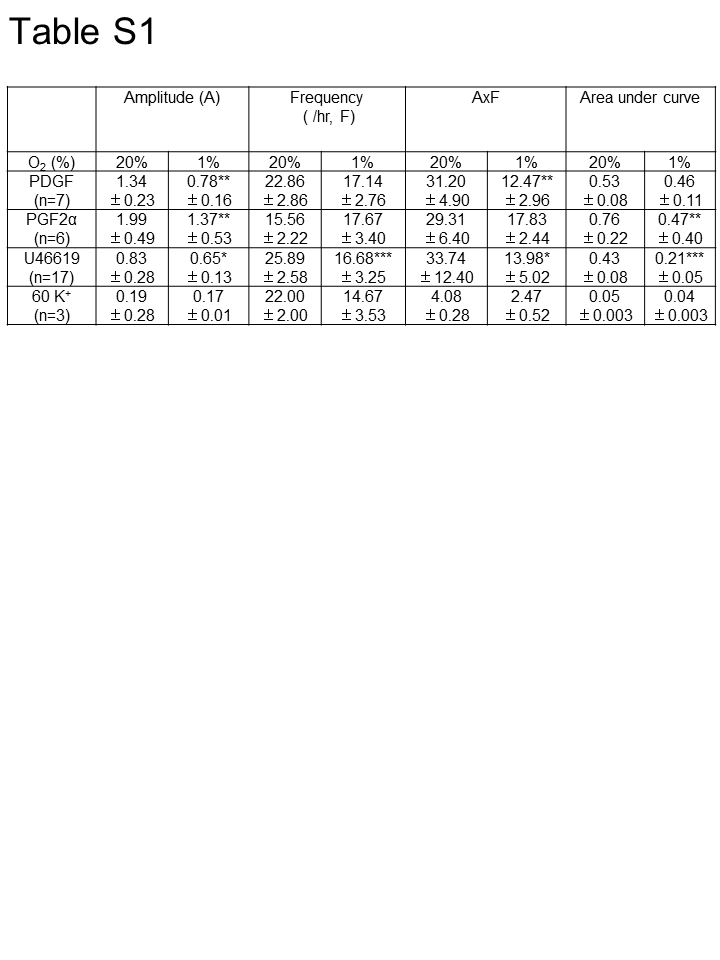

Supplement: Supplemental Information 10 — Effects of hypoxia (1% O2) on amplitude (A), frequency (F), AxF and Area under curve (AUC) of Ca2+ oscillations induced by PDGF (n = 7), PGF2 α (n = 6), U46619 (n = 17) and 60 K+ (n = 3). [file peerj-08-10344-s010.tif]

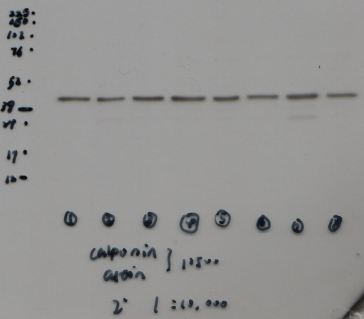

Supplement: Supplemental Information 47 [file peerj-08-10344-s047.jpg]

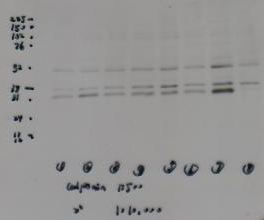

Supplement: Supplemental Information 48 [file peerj-08-10344-s048.jpg]

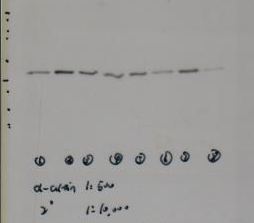

Supplement: Supplemental Information 49 [file peerj-08-10344-s049.jpg]
